# Causal graph-based analysis of genome-wide association data in rheumatoid arthritis

**DOI:** 10.1186/1745-6150-6-25

**Published:** 2011-05-18

**Authors:** Alexander V Alekseyenko, Nikita I Lytkin, Jizhou Ai, Bo Ding, Leonid Padyukov, Constantin F Aliferis, Alexander Statnikov

**Affiliations:** 1 Center for Health Informatics and Bioinformatics, New York University School of Medicine, New York, NY 10016, USA; 2 Department of Medicine, New York University School of Medicine, New York, NY 10016, USA; 3 Institute of Environmental Medicine, Karolinska Institutet, SE-171 77 Stockholm, Sweden; 4 Rheumatology Unit, Department of Medicine, Karolinska Institutet and Karolinska University Hospital Solna, SE-171 76 Stockholm, Sweden; 5 Department of Pathology, New York University School of Medicine, New York, NY 10016, USA; 6 Department of Biostatistics, Vanderbilt University, Nashville, TN, 37232, USA

## Abstract

**Background:**

GWAS owe their popularity to the expectation that they will make a major impact on diagnosis, prognosis and management of disease by uncovering genetics underlying clinical phenotypes. The dominant paradigm in GWAS data analysis so far consists of extensive reliance on methods that emphasize contribution of individual SNPs to statistical association with phenotypes. Multivariate methods, however, can extract more information by considering associations of multiple SNPs simultaneously. Recent advances in other genomics domains pinpoint multivariate causal graph-based inference as a promising principled analysis framework for high-throughput data. Designed to discover biomarkers in the local causal pathway of the phenotype, these methods lead to accurate and highly parsimonious multivariate predictive models. In this paper, we investigate the applicability of causal graph-based method TIE* to analysis of GWAS data. To test the utility of TIE*, we focus on anti-CCP positive rheumatoid arthritis (RA) GWAS datasets, where there is a general consensus in the community about the major genetic determinants of the disease.

**Results:**

Application of TIE* to the North American Rheumatoid Arthritis Cohort (NARAC) GWAS data results in six SNPs, mostly from the MHC locus. Using these SNPs we develop two predictive models that can classify cases and disease-free controls with an accuracy of 0.81 area under the ROC curve, as verified in independent testing data from the same cohort. The predictive performance of these models generalizes reasonably well to Swedish subjects from the closely related but not identical Epidemiological Investigation of Rheumatoid Arthritis (EIRA) cohort with 0.71-0.78 area under the ROC curve. Moreover, the SNPs identified by the TIE* method render many other previously known SNP associations conditionally independent of the phenotype.

**Conclusions:**

Our experiments demonstrate that application of TIE* captures maximum amount of genetic information about RA in the data and recapitulates the major consensus findings about the genetic factors of this disease. In addition, TIE* yields reproducible markers and signatures of RA. This suggests that principled multivariate causal and predictive framework for GWAS analysis empowers the community with a new tool for high-quality and more efficient discovery.

**Reviewers:**

This article was reviewed by Prof. Anthony Almudevar, Dr. Eugene V. Koonin, and Prof. Marianthi Markatou.

## Background

Genome-wide association studies (GWAS) are considered to be one of the primary tools for determining genetic links to disease. GWAS have been abundant in recent scientific research with more than 900 primary large-scale studies performed in the last 5 years. Each of these studies has genotyped at least 100,000 single nucleotide polymorphisms (SNPs) in cohorts that often exceed 1,000 subjects [1]. Overall, the reason for popularity of GWAS is the expectation that they will lead to discovery of SNPs implicated in disease and development of predictive models that can facilitate diagnosis, management, and treatment of disease.

Despite recent expansion of genome-wide association studies, methodologies for statistical analysis of the resulting data are still lagging behind. The most dominant paradigm for such analyses is focused on assessing contribution of individual SNPs to statistical association or risk of developing a phenotype [2-5]. Multivariate methods take a step forward by shifting the focus on how combining the individual SNP signals can help classify the phenotypes, and thus uncover additional evidence for possible genetic risk factors. Among multivariate techniques, Bayesian networks, kernel-based classifiers and multivariate regression are making their way as candidate new methodologies for the analysis of GWAS data [6-11].

**Figure 1:**
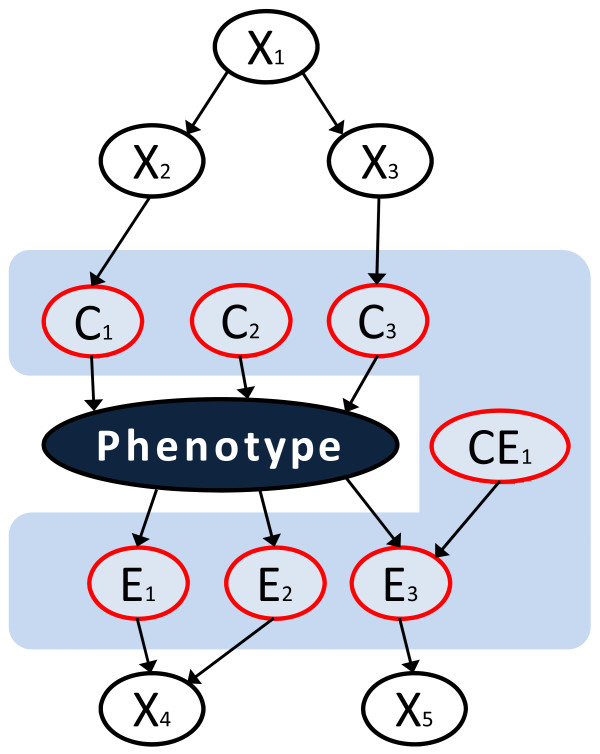
**Graphical representation of the local pathway concept**. The local pathway of the phenotype (shown with the ash blue colour) contains all its direct causes (C_1_, C_2_, C_3_), direct effects (E_1_, E_2_, E_3_), and direct causes of the direct effects (CE_1_). This is exactly the Markov boundary of the phenotype. Other variables (X_1_, X_2_, X_3_, X_4_, X_5_) do not belong to the local pathway. This definition of a local pathway ties in a theoretically rigorous manner causality with predictivity, since the Markov boundary is the smallest set of variables that contains the maximum predictive information about the phenotype that is contained in the data. Alternative definitions of the local causal pathway that exclude direct causes of the direct effects (the so-called "spouse variables", such as CE_1_) are also useful and specialized algorithms exist to infer them from data. In GWAS data, the two definitions coincide because of lack of spouse variables in GWAS designs.

Of particular relevance to the goals of GWAS are recent multivariate causal graph-based methods that are computationally efficient and can scale well to the dimensionality of GWAS data [12-14]. Unlike simple discovery of univariate associations, these methods can discover biomarkers (SNPs) in the local pathway of the phenotype (referred to as "local causal biomarkers", see Figure 1 for a graphical representation) under reasonably broad assumptions [12-14]. Local causal biomarkers constitute the Markov boundary and yield the highest accuracy predictions of the phenotype, while other biomarkers do not contribute additional predictive information beyond what is contained in the local causal ones [12-15]. In addition, the set of local causal biomarkers exhibits maximum parsimony, beyond which predictive accuracy is compromised [12-14,16].

Causal graph-based methods have been previously applied in a number of studies involving various high-throughput assays (e.g., microarray and proteomics) and phenotypes, yielding both highly accurate and parsimonious predictive models of disease as well as biomarkers implicated in disease mechanisms [12,14,17,18]. To the best of our knowledge, causal graph-based methods have not been previously applied to analysis of GWAS data.

In this paper we perform a case study for applicability of causal graph-based framework to analysis of GWAS data. We chose to apply the methodology to rheumatoid arthritis, a common autoimmune disease with unknown etiology [19,20], precisely because the genetics of this disease has been thoroughly studied in the last 20 years and there is a general consensus in the community about the major genetic determinants of this disease. Recent findings illuminated the importance of auto-antibodies to citrullinated protein/peptide antigens (ACPA), which could be routinely detected by ELISA using anti-cyclic citrullinated peptides (anti-CCP) and are now included as a diagnostic criteria for rheumatoid arthritis [21]. The anti-CCP positive subgroup of rheumatoid arthritis represents specific well-studied features regarding genetic and environmental risk factors [22,23], differs in clinical features and responsiveness to the treatment and, possibly, is pathogenetically distinct from the other forms of rheumatoid arthritis [20]. We focus our analysis specifically on the anti-CCP positive subgroup of rheumatoid arthritis using data from North American Rheumatoid Arthritis Cohort (NARAC) [3] and Swedish Epidemiological Investigation of Rheumatoid Arthritis (EIRA) cohort [22]. These data have been published and thoroughly analyzed using a wide range of univariate methods.

In the remainder of this paper, we describe the application of a state-of-the-art causal graph-based algorithm TIE* (which is an acronym for Target Information Equivalency) to anti-CCP positive rheumatoid arthritis GWAS data [12]. We cover all major steps of a typical study, including examining the data for potential biases, discovery of biomarkers, testing the robustness of the identified biomarkers, building predictive models and validating the results in independent data.

## Results and Discussion

### Patterns of missing data in NARAC cohort are not random with respect to the phenotype

On our initial examination of NARAC GWAS data we have noticed that, even after applying data completeness filtering (see Methods and Materials), many SNPs still contain a large number of missing genotype calls. As a matter of quality assurance, we have decided to test whether the patterns of missing genotype calls contain relevant information for predicting the phenotype. To do so, we modify the filtered dataset such that only information about presence or absence of each SNP genotype call is retained. In other words, each missing SNP call is encoded as "1" and all non-missing calls are encoded as "0". Following the cross-validation design of our study (see Methods and Materials), we use the training set of NARAC subjects to build a classification model to predict the phenotype based on all 490,073 SNPs. The resulting predictive model achieves an extremely high classification accuracy of 0.96 AUC in NARAC testing data (95% confidence interval: [0.95; 0.98] AUC) when classifying rheumatoid arthritis cases and controls.

This finding indicates that SNPs are not missing at random and there must be a pattern that may or may not be biologically meaningful. One plausible biological explanation is that the somatic modifications such as mutations and copy number variations are causing the genotyping platform to call missing values. Another plausible biological explanation of non-random missing SNP calls can be attributed to alternative haplotypes linked to the MHC locus. To provide evidence for this hypothesis, we perform a hyper-geometric test for enrichment of SNPs with missing genotype values significantly associated with rheumatoid arthritis in the MHC locus. Out of all 338,774 SNPs that have missing values, 17,379 (5.1%) have missing values significantly associated with rheumatoid arthritis according to *G*^2 ^test at 0.05 false discovery rate. However, out of all 945 SNPs with missing values in the MHC locus, 81 (8.6%) have missing values significantly associated with rheumatoid arthritis. Therefore, enrichment of SNPs at the MHC locus is statistically significant (p-value < 10^-5^).

Technological explanations of the association of missing SNP data patterns with the phenotype are possible as well (e.g., due to batch effects). Short of performing additional assays to evaluate significance of the non-random patterns of missing data, a legitimate approach to deal with these potentially biasing and misleading SNPs is to remove them from the analysis. In general, such a conservative approach may potentially eliminate some of the relevant SNPs from the input data and reduce overall predictive accuracy. However, an additional analysis conducted by taking into consideration information from SNPs with missing calls and discussed below, shows that removing such SNPs does not compromise predictive accuracy in our study. We therefore employ this conservative filtering throughout the present work.

### As few as five SNPs can accurately predict anti-CCP positive rheumatoid arthritis

**Figure 2:**
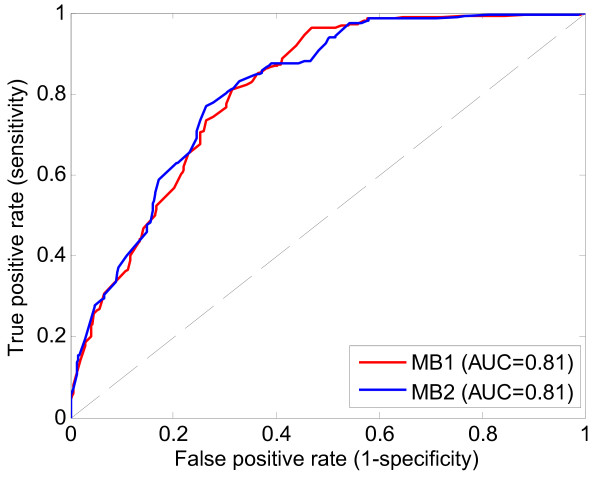
**ROC curves for the two causal graph-based predictive models applied to NARAC testing set**. Model denoted with "MB1" was fit using five SNPs from the first Markov boundary; model denoted with "MB2" was fit using five SNPs from the second Markov boundary.

Application of the causal graph-based method TIE* to the training set of NARAC subjects results in two five-SNP information equivalent Markov boundaries of the rheumatoid arthritis phenotypic response variable (denoting case and control status of the subjects). The two Markov boundaries jointly contain a total of six SNPs and have four SNPs in common (Table 1). SNP rs9275374 that is included in the first Markov boundary is substituted by SNP rs9275390 in the second Markov boundary due to these two SNPs exhibiting complete linkage disequilibrium (LD) in our dataset (R^2 ^= 1, see Table 2). We fit kernel ridge regression to both Markov boundaries to produce predictive models (molecular signatures) of anti-CCP positive rheumatoid arthritis. Each of these predictive models achieves 0.81 AUC (95% confidence interval: [0.78; 0.84] AUC) in the independent testing set of NARAC cohort (Figure 2).

**Table 1:** SNPs identified by the TIE* method in NARAC training set.

dbSNP ID	Markov boundary	Chromosome*		Minor allele frequency in NARAC cases	Minor allele frequency in NARAC controls	Gene name
		Number	coordinate			
***rs660895***	1,2	6	32,577,380	53%	19%	-
***rs6910071***	1,2	6	32,231,452	51%	20%	*C6orf10*
***rs9275390***	1	6	32,669,156	48%	25%	-
***rs3129871***	1,2	6	32,406,342	17%	37%	*HLA-DRA*
***rs9275374***	2	6	32,668,526	48%	25%	-
***rs12523624***	1,2	5	142,020,508	53%	48%	*FGF1*

These SNPs participate in the two Markov boundaries of the rheumatoid arthritis phenotypic response variable (denoting case and control status of the subjects).

* based on dbSNP and GRCh37 *Homo sapiens *Genome Build 37 version 1.

**Table 2:** Contingency tables for the two SNPs that are in complete linkage disequilibrium.

		*rs9275390*						
	
	NARAC cases	AA	AG	GG	NARAC controls	AA	AG	GG
***rs9275374***	**AA**	0	0	182	**AA**	0	0	66
	**AG**	0	468	0	**AG**	0	453	0
	**GG**	213	0	0	**GG**	662	0	0

An additional analysis where SNPs with missing genotype values are retained in the data results in a Markov boundary with 8 SNPs (Table S1 in [Additional File 1]) and exactly the same predictive accuracy in the NARAC testing set (0.81 AUC). Therefore, predictive accuracy is not affected by removing SNPs with missing genotype calls.

### Causal graph-based predictive modelling yields reproducible genetic biomarkers of rheumatoid arthritis

Five of the six SNPs that participate in the causal graph-based predictive models (rs660895, rs6910071, rs9275390, rs3129871, and rs9275374) map to *HLA-DR *locus at chromosome 6, which is a component of MHC II (major histocompatibility complex II). The alleles of this complex have been found to confer a predisposing effect, a neutral effect, or a protective effect in rheumatoid arthritis [24]. SNP rs12523624 maps to chromosome 5 within the *FGF1 *(Fibroblast Growth Factor-1) gene is not known to be associated with rheumatoid arthritis, however polymorphisms of this gene have been linked with psoriatic arthritis [25].

A recent meta-analysis in 5,539 rheumatoid arthritis cases and 20,169 controls over 6 cohorts [26] reveals that at least four out of the six identified SNPs retain their statistically significant univariate association with the phenotype (Table 3). Data for SNP rs9275374 is not present in the meta-analysis; thus we cannot assess its statistical significance. However, this SNP is in complete LD with SNP rs9275390 (Table 2) and is therefore likely to reproduce its univariate association in subjects outside NARAC cohort. Only one of the six SNPs, rs12523624, does not retain its univariate association in the meta-analysis (p-value = 0.3335). This SNP is also found to be statistically non-robust as detailed in the next section.

**Table 3:** Results of meta-analysis for 6 SNPs selected by the TIE* method.

dbSNP ID	Meta-analysis odds ratio	Odds ratio 95% confidence interval		P-value
***rs660895***	3.29	3.12	3.46	0
***rs6910071***	2.88	2.73	3.03	0
***rs9275390***	2.22	2.12	2.34	<10^-16^
***rs3129871***	0.51	0.48	0.53	<10^-16^
***rs12523624***	1.02	0.98	1.07	0.33346

SNP rs9275374 was not included in the meta-analysis because it was either not measured in some cohorts or not could not be imputed.

### Identified SNPs and predictive models are also statistically robust

**Figure 3:**
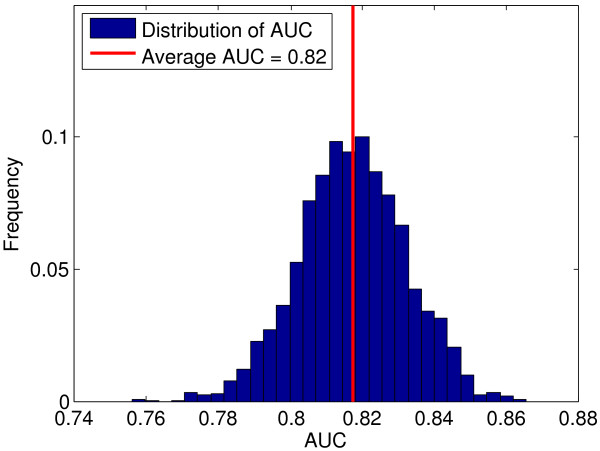
**Area under the ROC curve (AUC) for the causal graph-based predictive models developed in 1000 different random splits of NARAC data into training and testing sets**.

To assess statistical robustness of SNPs in the two Markov boundaries and of the associated predictive accuracies, we perform re-sampling analyses over 1000 different random splits of NARAC data into non-overlapping training and testing sets. Each training set is used for SNP selection by TIE* and fitting of predictive models with kernel ridge regression, while each testing set is only used for the final assessment of predictive accuracy. Over 1000 splits of the data, predictive models achieve an average accuracy of 0.82 AUC, with standard deviation 0.01 AUC (Figure 3). This demonstrates robustness of predictive accuracy of our models in the NARAC cohort. Similarly, five out of six SNPs reported in Table 1 show a high degree of statistical robustness and are selected in >50% of the training sets. Notably, 3 SNPs (rs660895, rs9275390, and rs9275374) are selected in all 1000 training sets. SNP rs12523624, which is not validated by the meta-analysis, also does not achieve significant robustness under re-sampling; it is selected in only the original training set. Table S2 in [Additional File 1] provides the list of all SNPs that are selected in at least 50 (5%) out of 1000 training sets in NARAC data.

### Many of the previously reported SNPs are statistically independent of the phenotype given SNPs discovered by the causal graph-based approach

**Figure 4:**
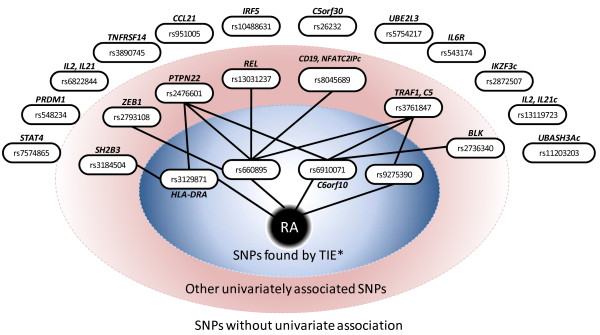
Previously known SNP associations become statistically independent of the phenotype conditioned on 4 SNPs discovered by TIE*. The phenotypic response variable is shown with black circle in the middle ("RA") and SNPs are shown with white ovals. SNPs that have a univariate association with the phenotype (according to *G*^2 ^test at significance level *α *= 5%) have a path to "RA". SNPs that become statistically independent of the phenotype given a subset of 4 SNPs found by TIE* (so-called "conditioning set") are connected with "RA" by indirect paths that go through SNPs in the corresponding conditioning set.

Several dozens of SNPs have known and previously verified association with rheumatoid arthritis. A recent study by Stahl et al. [26] provides a list of 47 such SNPs in Tables 2, 3, and 4 of their article. Out of these SNPs, only 20 are assayed in NARAC cohort using Infinium HumanHap550 platform. Out of the latter 20 SNPs, only one (rs6910071) is found by the causal graph-based method in our study. The remaining 19 SNPs are statistically independent of the phenotype (according to *G*^2 ^test at significance level *α *= 5%) conditioned on 4 Markov boundary SNPs in the MHC locus (see Figure 4). Therefore, these 19 SNPs do not carry for tested NARAC cohort any predictive information about rheumatoid arthritis beyond that provided by 4 MHC SNPs discovered by the causal graph-based approach. Note that SNPs rs2476601 (*PTPN22*) and rs3761857 (*TRAF1-C5*) are known to be strongly associated with rheumatoid arthritis from previous analyses. These SNPs require at least 3 SNPs from TIE* to be rendered conditionally independent of the phenotype.

### Causal graph-based predictive models generalize to Swedish subjects in EIRA cohort

We validate the NARAC-based predictive models of rheumatoid arthritis in independent data from EIRA cohort. Due to substantial differences in the subject recruitment protocols, we start by assessing the statistical differences between cases and controls in the two cohorts and possible implications for the model performance. We assess such differences by building and applying two classifier models trained to predict the cohort from which the subjects are derived (NARAC or EIRA) from SNP genotype data: (1) for cases and (2) for controls. The classifier for controls results in 0.53 AUC (95% confidence interval: [0.50; 0.55] AUC). Since the predictive accuracy of a random classifier (0.50 AUC) falls within the estimated confidence interval, we conclude that the controls in NARAC and EIRA cohorts are statistically comparable. However, the classifier for cases leads to 0.60 AUC (95% confidence interval: [0.58; 0.63] AUC), indicating that NARAC cases are statistically different from EIRA, however the magnitude of this difference is relatively small. This empirical analysis shows that the differences between NARAC controls and EIRA controls are smaller than between NARAC cases and EIRA cases. Thus, we do not anticipate perfect generalization of NARAC-based models to EIRA cases.

To control for the differences between EIRA and NARAC cohorts, we validate NARAC-based models in three independent datasets consisting of: (i) EIRA controls and NARAC testing set cases, (ii) EIRA cases and NARAC testing set controls, and (iii) EIRA cases and controls. We emphasize that in order to avoid biasing predictive accuracy the above validation datasets do not contain any subjects that are used for building NARAC-based models.

**Figure 5:**
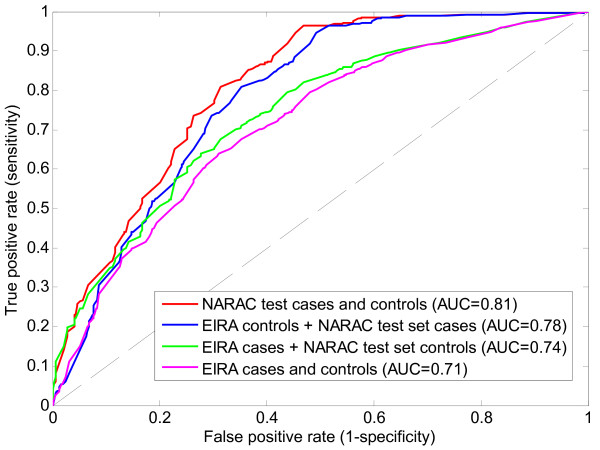
**ROC curves for validation of the causal graph-based predictive model of rheumatoid arthritis (that was developed in NARAC training set) in EIRA cohort**.

As shown in the previous section, two equivalent models (based on two Markov boundaries) developed in NARAC training set achieve predictive accuracy 0.81 AUC (95% confidence interval: [0.78; 0.84] AUC) in NARAC testing set. We further validate in EIRA data the model based on the first Markov boundary only (Table 1), since SNP rs9275374 is not genotyped in EIRA cohort. Figure 5 provides ROC curves for validation of the corresponding model in EIRA datasets. When this model is validated with EIRA controls and NARAC testing set cases, the obtained predictive accuracy of 0.78 AUC falls inside the 95% confidence interval for NARAC data only, which confirms the expected generalization of the model. Next, when the model is applied to EIRA cases and NARAC testing set controls, the resulting AUC is 0.74, which is lower than performance in NARAC data only. When the model is applied to EIRA cases and controls, its performance is 0.71 AUC. This decrease in performance is most likely a result of compounding of the differences in cases and controls of the two cohorts that were quantified earlier in this section.

**Figure 6:**
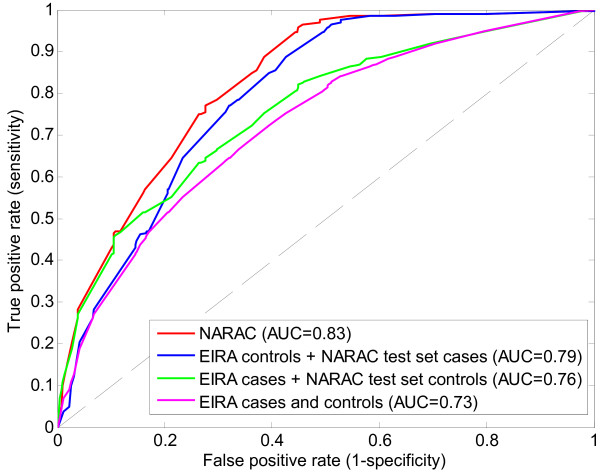
**ROC curves for validation of the modified causal graph-based predictive model of rheumatoid arthritis (without SNP rs12523624) in EIRA cohort**.

Using EIRA data we can also explore whether SNP rs12523624 (that does not belong to the MHC locus), which has been found to be statistically non-robust in NARAC and invalidated in the meta-analysis in several cohorts, is predictively essential for the causal graph-based model of rheumatoid arthritis. We build a modified model by removing SNP rs12523624 from the first Markov boundary and fitting kernel ridge regression to the remaining 4 SNPs in NARAC training data. Interestingly, the resulting model yields slightly better predictive accuracy than the original 5-SNP model (Figure 6). However, the observed differences are not statistically significant. Hence we conclude that the effect of SNP rs12523624 on predicting rheumatoid arthritis is small and this SNP is likely to be a false positive marker. However, we note that the results in Figure 6 may be slightly overoptimistic because both statistical robustness analysis and meta-analysis (that informed the removal of SNP rs12523624) utilize subjects used for validation of the model.

### Causal graph-based predictive models compare favourably to previously reported predictive techniques

The comparison of our results with prior efforts is facilitated by the fact that the same NARAC GWAS dataset has been previously used for predictive modelling of rheumatoid arthritis in the 16^th ^Genetic Analysis Workshop [27]. In the context of this workshop, Arshadi et al. applied gradient-boosting machine methodology to predict case-control status of subjects in NARAC cohort [28]. As can be seen in Figure 2 of their paper [28], predictive accuracy in the independent test set in NARAC does not exceed 0.77-0.78 AUC, which is slightly lower than the results of our causal graph-based models (0.81 AUC). Notably, Arshadi et al. achieves predictive accuracy greater or equal to 0.77 AUC only for models based on at least 5,000-10,000 SNPs which is four orders of magnitude larger than the number of SNPs in our causal graph-based models (with five SNPs only). In addition, it is worth briefly mentioning the results of Liang et al. [29] and Jeffries and Zheng [30] who have also analyzed NARAC dataset for predictive modelling but have used SNPs selected in the prior analyses of the same NARAC dataset. Such choice of SNPs is expected to yield overoptimistic estimates of predictive accuracy [31], and thus results of these papers should be treated with great caution. Although possibly biased, the previously reported results still correspond to lower predictive accuracy than the causal graph-based models: Liang et al. achieved a proportion of correct classifications equal to 76% and Jeffries and Zheng achieved 0.75 AUC. Finally, in contrast with our work, the computational methods used in prior studies to identify SNPs do not have a theoretical basis for causal interpretation.

*HLA *shared-epitope (SE) status has been suggested for predictive purposes; therefore, we also compare the accuracy of the causal graph-based predictive models (based on combination of five SNPs) to the predictive performance of the SE status that is recorded for all subjects in EIRA and NARAC cohorts independently of SNP data from GWAS. SE status is encoded as 1 when one of the following alleles is present: DRB1*0101, 0102, 0104, 0105, 0401, 0404, 0405, 0408, 0409, and 1001; and 0 otherwise. When applied to NARAC data, the performance of SE status is 76% sensitivity and 68% specificity. The performance of the causal graph-based model is very similar and not statistically different: it achieves 81% sensitivity at the same level of specificity and 70% specificity at the same level of sensitivity. When applied to EIRA data, the performance of SE status is 85% sensitivity and 50% specificity. The performance of the causal graph-based model is again very similar and not statistically different: it achieves 82% sensitivity at the same level of specificity and 44% specificity at the same level of sensitivity. Although our SNP-based model does not achieve a significant improvement in predictive accuracy over using the SE status at the corresponding levels of sensitivity and specificity, the SNP-based model is more informative and potentially more practical since it can operate at any desired level of sensitivity or specificity, unlike the binary SE status which is limited to a single level of sensitivity and specificity.

## Conclusions

Our study demonstrates the benefits of using causal graph-based methods for the analysis of GWAS data in anti-CCP positive rheumatoid arthritis. When applied to NARAC cohort, the causal graph-based method TIE* has discovered 6 SNPs (mostly from the MHC locus) in the local causal pathway of the phenotype that can be used to predict anti-CCP positive rheumatoid arthritis with the accuracy of 0.81 AUC. The predictive performance also generalizes reasonably well to the independent data from EIRA cohort. Finally, the SNPs identified by the TIE* method render all other previously known SNP associations conditionally independent of the phenotype in NARAC cohort. These findings, corroborated by comparison with the literature and expert knowledge of rheumatoid arthritis, suggest that TIE* allows for unravelling of the maximum amount of genetic information about the disease that is available in the data in a highly efficient and short discovery cycle.

## Methods

### Dataset description and pre-processing

NARAC dataset contains 863 cases (subjects with anti-CCP positive rheumatoid arthritis) and 1,181 controls (rheumatoid arthritis-free subjects), which are genotyped on the Illumina Infinium HumanHap550 platform for 545,080 SNPs [3] using whole blood as a source for genomics DNA. First, we apply pre-processing methods identical to the ones used in the original report on these data [3]. Specifically, we use the following SNP filtering steps/criteria: (1) SNP call must be present in >95% of subjects ("completeness filtering"); (2) minor allele frequency must be above 1%; and (3) SNP must not violate Hardy-Weinberg equilibrium at *α *= 10^-5^. The following number of SNPs is filtered in each of these steps, respectively: 18,718, 22,984 and 13,305, resulting in a dataset with 490,073 SNPs.

EIRA dataset contains 1,146 cases (subjects with anti-CCP positive rheumatoid arthritis) and 1,078 controls (rheumatoid arthritis-free subjects), which are genotyped on the Illumina Infinium HumanHap300 platform for 317,503 SNPs [3,32]. We have only selected the genotypes for SNPs selected in NARAC training data for validation purposes of the causal graph-based predictive models.

It is worthwhile to mention differences between EIRA and NARAC subjects. EIRA cases are incident rheumatoid arthritis cases (on average about 7 months after the first symptom onset, and registered at first encounter with a rheumatology specialist). They have been recruited from a whole population in mid and south of Sweden. Thus they are not biased by selection in referral systems and by severity and effects of treatment, but correspond to a rather representative sample of incident cases of rheumatoid arthritis in Sweden. On the other hand, NARAC cases have been obtained by referral from rheumatology clinics in various parts of the U.S., and are thus affected by referral patterns and response to initial treatment. EIRA controls have been randomly selected to represent the population in the areas of case recruitment and to match the cases by sex, age and area of residence. On the other hand, NARAC controls have been recruited from New York Cancer Project which possibly introduces selection bias [33]. Thus we conjecture that the differences between NARAC controls and EIRA controls are smaller than between NARAC cases and EIRA cases.

### Cross-validation design and methods for estimating accuracy of predictive models

To estimate accuracy of predictive models, we use hold-out cross-validation [34]. This design controls for SNP selection and predictive model bias and eliminates the possibility of overfitting [31,35,36]. First, NARAC data is split at random into non-overlapping training (with ~67% or 1366 subjects) and testing (with ~33% or 678 subjects) sets. The split retains the proportion of cases and controls. Then the testing set is put aside, while SNPs are selected in the training set and predictive models utilizing these SNPs are also developed in the training set. Finally, accuracy of the above predictive models is assessed in the testing set. In addition, we perform another validation of predictive accuracy using subjects from EIRA cohort.

As a metric of predictive accuracy, we use area under the ROC curve (AUC). This metric has larger statistical power to detect predictive signal than the commonly used proportion of misclassifications [37]. The ROC curve is the plot of sensitivity versus one minus the specificity for a range of continuous or discrete classification threshold values. AUC ranges from 0 to 1, with an AUC equal to 0 indicating the classifier opposite to the perfect, 0.5 representing a random (i.e., uninformative) classifier, and 1 representing perfect classification [38]. The 95% confidence intervals on AUC are obtained using the method by Delong et al. [39].

### Causal graph-based methods for selection of SNPs

We apply the TIE* method to the training data of NARAC for selection of SNPs that can be implicated in the development of rheumatoid arthritis. The method was introduced in [12] (a brief description of TIE* is also given in [Additional File 2]) to enable probabilistic modelling of multiple signatures of the disease. The method is based on Markov boundary induction, which formally connects biomarkers and the phenotype into a causal graph ("pathway") of the data generating process even when this pathway is not known *a priori *[16,40-42]. TIE* outputs all Markov boundaries of the phenotypic response variable, which are minimal sets of SNPs that render all other SNPs statistically independent of the phenotype [40]. Since each of the Markov boundaries corresponds to non-redundant biomarkers in a maximally predictive signature, TIE* addresses the phenomenon of multiplicity of molecular signatures and can facilitate separation of statistical instability in biomarker selection process from intrinsic information equivalency in the biological system [12].

Under the following four standard and sufficient causal discovery assumptions, Markov boundaries output by TIE* contain only SNPs that belong to the local pathway of the phenotype (i.e., all SNPs that are direct causes, direct effects, and direct causes of the direct effects of the phenotype, see Figure 1) or are statistically equivalent to these SNPs [12-15]: (i) adjacency faithfulness relaxed to allow for multiplicity of data-consistent local pathways [43-45]; (ii) causal Markov condition; (iii) correctness of statistical decisions, and (iv) causal sufficiency [14,15]. In non-technical terms, the first two assumptions mean that with the exception of empirical information equivalency relations between SNPs, there is a direct correspondence between data and directed acyclic data-generative graph in terms of statistical relations. Specifically, there is an edge between two SNPs if and only if they have non-zero association in the data conditioned on every subset of other SNPs. The third assumption means that determination of statistical (in)dependence relationships between SNPs in the population from the available data sample is correct. The fourth assumption means that every common cause of two or more measured SNPs is also measured in the dataset. We emphasize that these are only sufficient assumptions, and modern Markov boundary induction algorithms are robust to violations of the above assumptions [13,14].

The extent to which these sufficient assumptions hold or do not hold in GWAS data is currently unknown, however we provide below some facts that allowed us to conclude that these are reasonable assumptions for this type of data. With regards to the first assumption (relaxed faithfulness), prior research has established that non-faithful discrete probability distributions are extremely rare, and therefore it is reasonable to make this assumption here [46]. The second assumption (causal Markov condition) is a foundational assumption of Bayesian networks [47] and it is reasonable given empirical success of Bayesian networks in modelling SNP-SNP and SNP-phenotype relations, e.g. see [7,10,11,48]. The third assumption (correctness of statistical decisions) can be justified by the relatively large sample size in GWAS dataset. Finally, the fourth assumption (causal sufficiency) is reasonable because hundreds of thousands of SNPs are measured in GWAS study that map to or are linked with essentially all known genes in the human genome. On the other hand, we admit that the possibility of hidden confounders in GWAS data may challenge the causal sufficiency assumption. Some possible sources of hidden confounders in our study include inability of the employed genotyping platform to capture all genetic variability (for example, some essential SNPs or rare variants will never be in the dataset), unobserved mutation and recombination processes causing linkage disequilibrium, and SNPs with missing values that were removed from the analysis. Nevertheless, under the first, second, and third assumptions TIE* will not falsely remove true direct causes of the phenotype from its output as long as these SNPs are included in the input dataset. Thus, the SNPs identified by TIE* can include direct causes of the phenotype as well as distant causes or confounders in high linkage disequilibrium with true direct causes.

We run TIE* using GLL (Generalized Local Learning) as the base Markov boundary induction algorithm [13,14]. This choice of the base algorithm is motivated by both its empirical performance in biomedical high-throughput data and its theoretical properties [13,14]. The main operating principle of the GLL algorithm is elimination of SNPs that are irrelevant for the phenotype (i.e., do not belong to its local pathway) via tests of statistical independence conditioned on various subsets of the tentative local pathway set of SNPs [14]. GLL is run with the *G*^2 ^test at significance level *α *= 5% and with maximum conditioning set size parameter *max-k *= 3. We also require at least 5 samples per cell in the contingency tables for the *G*^2 ^test. The upper bound on the size of the conditioning set is justified by empirical performance in a variety of data distributions [13,14], as well as by sample size limitations in our data. Specifically, given (i) a sample size of 1366 subjects in the training dataset, (ii) the requirement of 5 samples per cell in contingency tables, (iii) a binary response variable, and (iv) and typically ternary SNP variables, the maximum size of a conditioning set used to establish independence relationships between SNPs and the response variable is ⌊log_3_(1366/(5·2·3))⌋ = #3 SNPs (where "⌊.⌋" denotes the integer part).

The GLL algorithm has a strong built-in capacity to control against false positives (i.e., it is very unlikely that the algorithm will output SNPs that do not belong to the local pathway of the phenotype), as we have discussed in depth in [13] and illustrated under a variety of high-dimensional settings using a well-controlled experimental design. The quality of statistical decisions for exclusion of irrelevant SNPs from the output of GLL is determined by the combined, or effective, significance threshold, which decreases at most exponentially with the number of statistical independence tests applied to each irrelevant SNP. Therefore, the combined probability of not eliminating irrelevant SNPs and thereby admitting false positives into the output of GLL is exceedingly small. On the other hand, the algorithm preserves a high combined power of statistical decisions and is therefore robust to falsely removing SNPs that truly belong to the local pathway of the phenotype. This in turn is achieved by (i) using correlated tests of statistical independence due to overlapping conditioning sets, (ii) fixing *max-k *parameter to a small constant (e.g., 3), and (iii) requiring at least 5 samples per cell in the contingency tables. The latter two parameters restrict the number of conditional independence tests performed by GLL and balance the number of false positives and false negatives that may appear in the output of the algorithm.

### Predictive modelling methods

After selecting SNPs with TIE*, we apply kernel ridge regression to build predictive models of rheumatoid arthritis using the NARAC training data [49,50]. We chose kernel ridge regression because it is one of the state-of-the-art supervised machine learning methods that is robust to high-dimensional data, can model highly non-linear relations between genotype and phenotype, and employs regularization to avoid overfitting [49,50]. In addition, the kernel ridge regression algorithm requires very little time for training of the classifier model (on the order of a few seconds in NARAC data), which allows to parameterize it efficiently by cross-validation. Kernel ridge regression is applied with the radial basis kernel function *K*(*x, y*) = exp(-*γ*||*x *- *y*||^2^). The choice of this kernel function was based on preliminary cross-validation experiments that indicated its higher classification performance in NARAC data. The regression ridge *λ *and kernel width parameter *γ *are determined by 10-fold cross-validation in the training set of NARAC data [51-53]. The above parameters are optimized over values of *λ *∈ {10^-10^, 10^-8 ^, ..., 1} and values of , where *n *denotes the number of SNPs participating in the model.

## Competing interests

The authors declare that they have no competing interests.

## Authors' contributions

AVA, AS and CFA conceived, designed and lead this study; they interpreted the results jointly with NIL, BD, LP and JA; NIL and JA performed computational experiments and analyses using causal graph-based methods invented by AS and CFA. All authors wrote the paper. AVA and AS contributed equally to this study. All authors read and approved the final manuscript.

## Reviewers' comments

### Reviewer #1

#### Reviewer's comments

The present paper investigates the applicability of the causal graph-based method TIE* to the analysis of GWAS data. The following comments must be addressed in the revision of the paper.

1) Causal graph-based methods rely on certain assumptions. The authors must discuss in the manuscript these assumptions and present convincing evidence that these assumptions are justified in the context of their case-study. How are these assumptions justified in the context of the paper?

2) Background section, in the 6th paragraph it is stated that "In the remainder of this paper, we describe the application of the state-of-the-art causal graph-based algorithm TIE* to.....". There are several issues that need to be addressed here. A) The authors need to include a succinct, yet informative, description of the TIE* algorithm. Paper references is not sufficient, this is supposed to be a self-contained piece of work. What are the strengths and limitations of the algorithm? Why is it appropriate to use it in this case study? And what are the important points of its implementation? B) Abbreviations should **never **be used without **first **defining those. What does TIE* stand for? All abbreviations must be defined first, then, they can be used.

3) Section entitled "Identified SNPs and predictive models are also statistically robust", first paragraph. A) It is stated in the manuscript that 100 different random splits of NARAC data are used and Figure 3 presents the distribution of AUC; this distribution is markedly skewed indicating that perhaps the number of splits is too small. Can the authors offer a rule that can be used to decide the number of splits in any given situation? It appears to me that the selection of this number is intimately related to the error of the classifier. A discussion of this point is necessary. B) What are the sizes of the training and test sets used? C) Kernel ridge regression was used; however, no intuition was given as to why this is appropriate. Also, what types of kernels were used and why?

4) Section entitled "Causal graph-based methods for selection of SNPs", first paragraph. It is stated that "Under fairly broad assumptions, Markov boundaries contain.....". A discussion that justifies these assumptions in this context must be presented. In the second paragraph it is stated that the maximum conditioning set parameter was 3. Why was this number selected? Discuss.

5) TABLES: In Table 3 results for 6 SNPs are presented. Among the different p-values reported there are p-values with ridiculous exponents, i.e. ten to the power -216 or -158. This does not make any scientific sense! What is the difference between these p-values and the ones equal to 0? This merely reflects the accuracy of the computer on which the analysis was carried out and provides no information about the underlying significance.

#### Authors' response

The point-by-point response follows:

1) The revised manuscript provides a detailed description and justification of the assumptions of causal graph-based methods used in this study. Please see second and third paragraphs of the sub-section "Causal graph-based methods for selection of SNPs".

2. A) We added the description of the TIE* algorithm to the Additional File 2 and also revised the algorithm description in the manuscript, please see first paragraph of the sub-section "Causal graph-based methods for selection of SNPs".

2. B) We explained all abbreviations in text when first referenced.

3. A) The smoothness of the distribution is ultimately dependent on the dataset sample size, classifier error, number of splits, and possibly other factors. The nature of this dependency is currently unknown and has not been quantified in high-dimensional biomedical data, such as GWAS. Therefore, per reviewer recommendation, we increased the number of splits to 1,000 that leads to a more smooth empirical distribution. Please see sub-section "Identified SNPs and predictive models are also statistically robust" and Figure 3 in the revised manuscript.

3. B) The sizes of the training and testing sets are now stated in the updated sub-section "Cross-validation design and methods for estimating accuracy of predictive models."

3. C) The justification for use of kernel ridge regression is provided in the revised manuscript, please see sub-section "Predictive modelling methods". This sub-section further includes the rationale for using radial basis function kernel which was used for our study.

4) Please see response to point 1) concerning causal discovery assumptions. We set the maximum conditioning set size parameter *max-k *= 3 primarily because of the sample size limitations as described in the fourth paragraph of sub-section "Causal graph-based methods for selection of SNPs" in the revised manuscript.

5) We agree with the reviewer, and corrected Table 4 accordingly.

### Reviewer #2

#### Reviewer's comments

The authors develop a SNP based biomarker for Rheumatoid Arthritis (RA) using data from the North American Rheumatoid Arthritis Cohort (NARAC) GWAS. This is developed using a causal graph model TIE* (citation [10], Statnikov and Aliferis). The biomarker is reported to have no worse predictability, using ROC curves, than a number of other reported alternatives, but with greater interpretability as a causal model (the authors find a relatively small set of SNPs which appear to account for all phenotype association). It is validated with a separate data set, where its performance is found to be approximately reproducible.

The methodology seems sound, with careful attention paid to issues of data quality, and represents and interesting case study in biomarker development. I have two concerns:

1) The authors correctly point out that much analysis of SNP association is confined to univariate analysis, but this comment is perhaps most relevant to the clinical literature, and even here it is not uncommon to use multivariate regression with second order interaction terms. I believe the literature on multivariate methods for SNP analysis is more extensive than the article suggests, and I also believe that Bayesian networks have been used in this application (eg Rodin SA, Boerwinkle E (2005) Mining genetic epidemiology data with Bayesian networks I: Bayesian networks and example application (plasma apoE levels) Bioinformatics 21(15): 3273-3278.)

2) An important aspect of this study is the use of causal models, which are able to infer conditional independence. This allows certain types of conclusions, for example, that the phenotype may be independent of a SNP conditionally on a specific SNP set G. This in turn means that that SNP contains no information regarding the phenotype not already contained in G. My understanding is that this is a consequence of the topology of the graphical model, as illustrated by the authors. This raises some concerns regarding goodness of fit. A causal model is designed precisely to detect conditional independence structure, expressible by the topology. Most fitting algorithms have some control for overfitting (in this case, usually expressed as spurious connectivity). It is also possible, therefore, to infer spurious conditional independence through excessive penalization of connectivity, and an objective balance is very hard to achieve. Is there any way, given the important of this issue in the manuscript, to separately validate those reported findings of conditional independence?

Minor corrections:

3) P5 L2 "an independent data <set>"

4) P11 A number of citations are not completely formatted.

5) Perhaps more description of the TIE*method would be helpful

#### Authors' response

The point-by-point response follows:

1) The revised manuscript contains expanded literature review about usage of multivariate methods for predictive analysis of SNP data. This includes the citation mentioned by the reviewer. Please see second paragraph of the revised "Background" section.

2) The discussion of false positives and false negatives in the output of the employed causal discovery algorithm is provided in the last paragraph of sub-section "Causal graph-based methods for selection of SNPs". One can also validate all found conditional independence relations in independent data. In the case of our study, we were provided only with a small set of SNPs for the independent validation dataset (EIRA), thus we cannot validate all conditional independence relations found in the NARAC dataset.

3) We corrected this issue in the revised manuscript.

4) We corrected citations and double-checked their formatting.

5) We added the description of the TIE* algorithm to the Additional File 2 and also revised the algorithm description in the manuscript, please see first paragraph of the sub-section "Causal graph-based methods for selection of SNPs".

### Reviewer #3: Dr. Eugene V. Koonin

#### National Center for Biotechnology Information, NIH, Bethesda, Maryland, United States

This reviewer provided no comments for publication.

## Supplementary Material

Additional file 1 Supplementary tables with SNPs selected by the TIE* method. Click here for file

Additional file 2 Description of the TIE* method. Click here for file
