# Supplementary material for: Causal graph-based analysis of genome-wide association data in rheumatoid arthritis
Source: Biol Direct. 2011 May 18;6:25. doi: 10.1186/1745-6150-6-25 (PMC3118953; doi:10.1186/1745-6150-6-25)
Supplement: Additional file 1 — Supplementary tables with SNPs selected by the TIE* method. [file 1745-6150-6-25-S1.DOCX]

# Additional File 1

**Table S1:** SNPs identified by the TIE* method in NARAC training set, when all SNPs with missing genotype values are retained in the dataset and missing genotype calls are ignored during computation of independence test statistics.

| **dbSNP ID** | **Chromosome*** | | | **Minor allele frequency in NARAC cases** | **Minor allele frequency in NARAC controls** | | **Gene name** | |  |
| --- | --- | --- | --- | --- | --- | --- | --- | --- | --- |
|  | **Number** | **Coordinate** | |  |  |  |  |  |  |
| ***rs2844509*** | 6 | | 31,618,903 | 15% | | 26% | | *BAT1* | |
| ***rs2395175*** | 6 | | 32,513,004 | 48% | | 15% | | - | |
| ***rs2516049*** | 6 | | 32,678,378 | 42% | | 31% | | - | |
| ***rs9275595*** | 6 | | 32,789,333 | 46% | | 22% | | - | |
| ***rs9409575*** | 9 | | 94,350,574 | 38% | | 31% | | - | |
| ***rs10849851*** | 12 | | 120,059,364 | 2% | | 4% | | *P2RX7* | |
| ***rs17546468*** | 13 | | 88,088,934 | 6% | | 7% | | - | |
| ***rs1182531*** | 20 | | 57,826,397 | 15% | | 22% | | *PHACTR3* | |
| ^*^ based on dbSNP and GRCh37 *Homo sapiens* Genome Build 37 version 1. | | | | | | | | |  |

**Table S2:** SNPs most frequently selected by TIE* in 1,000 different random splits of NARAC data into training and testing sets. Each of SNPs reported in this table was selected in at least 5% of splits.

| **dbSNP ID** | **Fraction of splits** | **Chromosome***  **Number Coordinate** | | **Gene name** | |
| --- | --- | --- | --- | --- | --- |
| ***rs660895*** | 100% | 6 | 32,577,380 | | - |
| ***rs9275374*** | 100% | 6 | 32,668,526 | | - |
| ***rs9275390*** | 100% | 6 | 32,669,156 | | - |
| ***rs6910071*** | 93% | 6 | 32,231,452 | | *C6orf10* |
| ***rs3129871*** | 57% | 6 | 32,406,342 | | *HLA-DRA* |
| ***rs2900180*** | 24% | 9 | 123,706,382 | | - |
| ***rs9268528*** | 23% | 6 | 32,331,315 | | - |
| ***rs3817963*** | 15% | 6 | 32,316,304 | | *BTNL2* |
| ***rs9275601*** | 10% | 6 | 32,606,072 | | - |
| ***rs1953126*** | 8% | 9 | 123,640,500 | | *PHF19* |
| ***rs10760130*** | 8% | 9 | 123,701,990 | | - |
| ***rs3763309*** | 6% | 6 | 32,324,176 | | *BTNL2* |
| ***rs12203592*** | 5% | 6 | 396,321 | | *IRF4* |
| ***rs4424066*** | 5% | 6 | 32,302,653 | | - |
| ^*^ based on dbSNP and GRCh37 *Homo sapiens* Genome Build 37 version 1. | | | | | |
