# Supplementary material for: Causal graph-based analysis of genome-wide association data in rheumatoid arthritis
Source: Biol Direct. 2011 May 18;6:25. doi: 10.1186/1745-6150-6-25 (PMC3118953; doi:10.1186/1745-6150-6-25)
Supplement: Additional file 2 — Description of the TIE* method. [file 1745-6150-6-25-S2.DOCX]

# Additional File 2

The most general description of the steps in the TIE* method [1] is provided in Figure S1. TIE* is a generative method that is instantiated differently for different distributions (an example of an instantiated TIE* method for GWAS data analysis is provided in Figure S2).

As shown in Figure S1, the following inputs are provided: (i) a dataset D (a sample drawn at random from distribution P) for variables **V**, including a response variable *T*; (ii) a single Markov boundary induction method X; (iii) a strategy Y to generate subsets of variables that have to be removed from **V** to indentify new Markov boundaries of *T*; and (iv) a criterion Z to verify Markov boundaries of *T*. The input components X, Y, and Z are selected to be suitable for the distribution in hand and should satisfy admissibility rules stated in Figure S3 in order for the method to be correct. The TIE* method when the admissibility requirements hold is guaranteed theoretically to output all Markov boundaries of *T* (i.e., all maximally accurate and non-redundant predictive models and predictor variable sets that participate in those models) [2].

In step 1, the TIE* method uses a single Markov boundary method X to learn a Markov boundary **M** of *T* from data D for variables **V** (i.e., in the "original" distribution). Then **M** is output in step 2. In step 3, an iterative loop is initiated. In step 4, the TIE* method applies the strategy Y to generate a subset **G** whose removal may lead to identification of a new Markov boundary of *T*. Next, in step 5 the Markov boundary method X is applied to a modification of dataset D in which the subset of variables **G** has been removed (this is referred to as an "embedded" distribution), resulting in a Markov boundary **M***_new_* in the embedded distribution. In step 6, if **M***_new_* is also a Markov boundary of *T* in the original distribution according to criterion Z, then **M***_new_* is output. The loop in steps 3-7 is repeated until all subsets **G** generated by strategy Y have been considered. The TIE* method outputs all and only Markov boundaries M of *T* as long as the admissibility criteria of Figure S3 hold [2].

| Generative method TIE*  Inputs:   - dataset D (a sample of distribution P) for variables **V**, including a response variable *T*; - Markov boundary method X; - strategy Y to generate subsets of variables that have to be removed to identify new Markov boundaries of *T*; - criterion Z to verify Markov boundaries of *T*.   Output: all Markov boundaries (i.e., maximally accurate and non-redundant signatures) of *T*.   1. Use method X to learn a Markov boundary **M** of *T* from data D for variables **V** (i.e., in the "original" distribution) 2. Output **M** 3. Repeat 4. Use strategy Y to generate a subset **G** whose removal may lead to identification of a new Markov boundary of *T* 5. Use method X to learn a Markov boundary **M***_new_* of *T* from data D for variables **V \ G** (i.e., in the "embedded" distribution) 6. If **M***_new_* is a Markov boundary of *T* in the original distribution according to criterion Z, output **M***_new_* 7. Until all subsets **G** generated by strategy Y have been considered. |
| --- |

**Figure S1:** Generative method TIE*.

| An example of instantiated method TIE* for GWAS data analysis  Inputs: GWAS dataset D for SNPs **V**, including a response variable *T*;  Output: all Markov boundaries (i.e., maximally accurate and non-redundant signatures) of *T*.   1. Use method GLL to learn a Markov boundary **M** of *T* from data D for SNPs **V** (i.e., in the "original" distribution) 2. Output **M** 3. Repeat 4. Generate the smallest subset **G** of the so far discovered Markov boundaries of *T* such that (i) it was not considered in the previous iteration of the method, and (ii) it does not properly include any subset that was generated in the previous iteration of the method when **M***_new_* was found not to be a Markov boundary of *T* 5. Use method GLL to learn a Markov boundary **M***_new_* of *T* from data D for SNPs **V \ G** (i.e., in the "embedded" distribution) 6. If each SNPs in **M** is statistically independent of *T* conditioned on **M***_new_*, then **M***_new_* is a Markov boundary of *T* in the original distribution and it is output by the method 7. Until no subset **G** can be generated in step 4. |
| --- |

**Figure S2:** An example of instantiated method TIE* for GWAS data analysis.

| Admissibility rules for inputs X, Y, Z of the TIE* method   1. The Markov boundary discovery method X correctly identifies a Markov boundary of *T* both in the original distribution (i.e., for variables **V**) and in the embedded distribution after removing an arbitrary set of variables. 2. The strategy Y to generate subsets of variables is complete, i.e. it will generate every subset **G** that is needed to be removed from **V** to identify every Markov boundary of *T*. 3. The criterion Z can correctly identify that **M***_new_* is a Markov boundary of *T* in the original distribution. |
| --- |

**Figure S3:** Admissibility rules for inputs X, Y, Z of the TIE* method.

# References

1. Statnikov A, Aliferis CF: **Analysis and Computational Dissection of Molecular Signature Multiplicity**. *PLoS Computational Biology* 2010, **6:**e1000790.

2. Statnikov A: **Algorithms for Discovery of Multiple Markov Boundaries: Application to the Molecular Signature Multiplicity Problem.**: Ph.D.Thesis, Department of Biomedical Informatics, Vanderbilt University; 2008.
